# Impact of inferior pulmonary ligament dissection versus preservation during thoracoscopic upper lobectomy: a retrospective comparative analysis

**DOI:** 10.3389/fsurg.2026.1812714

**Published:** 2026-04-20

**Authors:** Xinhe Huang, Zheng Zhu, Baisheng Xie, Kaifei Chen, Yue Xie, Yi Zhu

**Affiliations:** 1 Department of Obstetrics and Gynecology, The Second Affiliated Hospital of Zhejiang Chinese Medical University, Hangzhou, China; 2 Department of Cardiothoracic Surgery, Hangzhou TCM Hospital Affiliated to Zhejiang Chinese Medical University, Hangzhou, China

**Keywords:** bronchial angle, computed tomography, inferior pulmonary ligament, lung cancer, lung function, lung volume, thoracoscopic upper lobectomy

## Abstract

**Background:**

The clinical benefit of dividing the inferior pulmonary ligament (IPL) during video-assisted thoracoscopic upper lobectomy (TUL) for early-stage lung cancer remains controversial. This study aimed to evaluate the association between IPL division during TUL and postoperative clinical outcomes.

**Methods:**

We retrospectively analyzed 95 patients who underwent TUL between December 2020 and June 2025. Patients were assigned to an IPL-preservation group (Group P) or an IPL-division group (Group D). Group P included 50 patients (31 right-sided and 19 left-sided procedures), and Group D included 45 (29 right-sided and 16 left-sided procedures). Postoperative outcomes—including operative time, intraoperative blood loss, duration of postoperative air leak, chest tube duration, length of postoperative hospital stay, and changes in bronchial angle, lung volume, pulmonary function, and cough severity—were compared between groups. Bronchial angle and lung volume were measured using three-dimensional (3D) reconstructed chest computed tomography (CT) images. Cough severity and cough-related quality of life before and after surgery were assessed using the Mandarin Chinese version of the Leicester Cough Questionnaire (LCQ-MC).

**Results:**

Baseline characteristics were comparable between groups, with no statistically significant differences (all *P* > 0.05). IPL division was associated with a greater degree of postoperative bronchial angle change after left-sided surgery, reaching borderline significance at 3 months (68.1 ± 7.2° vs. 78.1 ± 7.8°, *P* = 0.046) and poorer 6-month outcomes, including smaller lung volume (3615 ± 475 mL vs. 3392 ± 489 mL, *P* = 0.027), a trend towards lower FEV1% (73.04 ± 9.36 vs. 69.06 ± 10.11, *P* = 0.049), lower DLCO (80.82 ± 10.35 vs. 76.06 ± 11.08, *P* = 0.033), and lower total LCQ-MC score (17.70 ± 1.72 vs. 16.98 ± 1.69, *P* = 0.042). No significant between-group differences were observed for other endpoints.

**Conclusions:**

IPL division did not demonstrate a clear benefit over IPL preservation. The findings suggest that IPL division may be associated with reduced postoperative lung volume, impaired recovery of diffusing capacity, greater displacement of the residual bronchus, and more severe chronic cough. However, particularly for outcomes with marginally significant P values, these results should be interpreted with caution due to multiple comparisons. These conclusions are hypothesis-generating and require confirmation in larger, prospective studies.

## 1 Introduction

Lung cancer remains a major global public health challenge and is the leading cause of cancer-related mortality worldwide (1, 2). Non-small cell lung cancer (NSCLC) accounts for approximately 80% of all lung cancer cases (2). Over the past few decades, video-assisted thoracoscopic surgery (VATS) has emerged as the standard minimally invasive approach for pulmonary lobectomy, offering reduced postoperative pain, shorter hospital stays, and oncologic outcomes comparable to those of open thoracotomy (3, 4).

As a pleural reflection distal to the lung hilum, the inferior pulmonary ligament (IPL) is formed by the apposition of parietal and visceral pleura and serves as an auxiliary stabilizing structure for the lower lobe (5). More than half of lung cancers arise in the upper lobes, particularly the right upper lobe (6–8). Whether IPL division is necessary during upper lobectomy remains debated. Recently, Campisi et al. conducted a multicenter matched cohort study evaluating the impact of inferior pulmonary ligament resection during upper lobectomy, providing additional evidence on postoperative outcomes and highlighting the ongoing controversy regarding IPL management (9). Nevertheless, detailed long-term anatomical volumetry, serial pulmonary function testing, and patient-reported cough outcomes were not reported, leaving important questions regarding functional recovery unanswered.

Traditionally, IPL division during upper lobectomy has been performed to facilitate lower-lobe re-expansion and reduce residual intrathoracic dead space, with the goal of decreasing postoperative pleural fluid accumulation (10, 11). However, robust evidence demonstrating clinically meaningful benefits of this practice is lacking. In the absence of a standardized consensus, intraoperative management of the IPL varies across surgeons and institutions (12). In addition to mechanical stabilization, the IPL may have physiological roles, potentially contributing to pleural fluid secretion and reabsorption (13). During upper lobectomy, IPL division has been reported to be associated with complications including bronchial stenosis, atelectasis, bronchial obstruction, sputum retention, and pneumonia (12, 14).

Therefore, we sought to contribute to the existing literature by comparing clinical outcomes between IPL preservation and IPL division during thoracoscopic upper lobectomy.

## 2 Methods

### 2.1 Patient selection

This study included 95 consecutive patients who underwent upper lobectomy for lung cancer between December 2020 and June 2025 at the Department of Thoracic Surgery, Hangzhou Traditional Chinese Medicine Hospital for Adults. Patients were divided into two groups according to IPL management: one group underwent IPL division (Group D), whereas the other group underwent IPL preservation (Group P).

### 2.2 Surgical technique

For patients with a significant air leak lasting more than 3 days, chemical pleurodesis was performed. This involved instillation of 50 mL of 50% glucose solution into the pleural cavity via the chest tube. The tube was then clamped for 60 min, during which the patient was repositioned regularly to facilitate distribution of the sclerosant. The tube was subsequently reconnected to suction.

All lung resections were performed by two board-certified thoracic surgeons experienced in independently performing thoracoscopic procedures. In accordance with International Association for the Study of Lung Cancer (IASLC) and European Society of Thoracic Surgeons (ESTS) guidelines, systematic lymph node dissection was performed in each operation (15). All patients received general anesthesia with double-lumen endotracheal intubation and one-lung ventilation. All procedures were performed using a utility incision with an additional observation port, and a fissure-based technique was used for thoracoscopic lobectomy. An endoscopic stapler was used to transect hilar structures, including pulmonary vessels and the bronchus. Absorbable polyglycolic acid felt (Neoveil; Guangzhou, China) was routinely applied to cover the bronchial stump and resection margins. For right-sided tumours, stations 2R, 4R, 7, 10R, and 11R were dissected when feasible. For left-sided tumours, stations 5, 6, 7, 10L, and 11L were dissected when feasible. In the IPL-preservation group, lobe-specific systematic nodal dissection was performed (1). However, when station 9 lymph node involvement was evident or strongly suspected, station 9 nodes were removed without dividing the IPL.

Two thoracic drainage tubes were placed at the end of the procedure under direct thoracoscopic visualization. A 26-Fr chest tube was inserted through the observation port at the seventh or eighth intercostal space along the mid-axillary line. The distal tip was positioned approximately 2–3 cm below the apex of the pleural cavity. A smaller 16-Fr catheter (with a puncture needle) was inserted at the ventral edge of the incision, with the distal tip positioned in the posterior mediastinum near the diaphragm. All drains were connected to a digital drainage system that continuously monitored air-leak rate and drainage volume, with real-time data displayed on a monitor. Significant air leak was defined as an air-leak rate >400 mL/min persisting for more than 3 days.

Chest tube removal was determined based on a comprehensive clinical assessment. The 26-Fr chest tube was removed when the air-leak rate was <100 mL/min over 24 h and chest radiography confirmed adequate lung re-expansion, defined as the highest point of the expanded lung reaching or exceeding the upper margin of the second posterior rib, with no residual extrapulmonary air space. The 16-Fr catheter was removed when the drainage volume was <200 mL within 24 h and thoracic ultrasound demonstrated no or minimal pleural effusion. All patients were extubated in the operating room after surgery.

### 2.3 Data collection

#### 2.3.1 Inclusion criteria

The inclusion criteria were as follows: (1) histologically confirmed non-small cell lung cancer; (2) tumor located in the upper lobe of either lung; and (3) treatment with video-assisted thoracoscopic surgery.

#### 2.3.2 Exclusion criteria

The exclusion criteria were as follows: (1) non-anatomical resections performed via VATS; (2) VATS anatomical segmentectomy; (3) VATS lower lobectomy, middle lobectomy, or bilobectomy; (4) VATS lobectomy combined with chest wall or diaphragmatic resection; (5) bronchial or vascular sleeve resection or a history of ipsilateral thoracic surgery; (6) conversion to thoracotomy; (7) receipt of neoadjuvant chemotherapy or thoracic radiotherapy; (8) prior breast cancer treatment or surgery for malignancies other than NSCLC; (9) pre-existing cavitary tuberculosis, bronchiectasis, or asthma, or marked cough symptoms; (10) (9) Diagnosis based solely on cytology without histological confirmation;and (11) missing or incomplete clinical data.

#### 2.3.3 Evaluation of perioperative outcomes

All enrolled patients were followed for at least 6 months after surgery. Early complications were defined as events occurring within 14 days after surgery, including atelectasis, pulmonary infection, atrial fibrillation, pleural effusion, and subcutaneous emphysema. Subcutaneous emphysema was recorded only when clinically significant, defined as air accumulation extending beyond the incision site and accompanied by palpable crepitus or swelling. Diagnosis was primarily based on physical examination and confirmed by chest x-ray to assess the extent of air. Focal, asymptomatic air confined to the incision edge was excluded from the analysis. Postoperative atelectasis was assessed based on imaging findings. Only segmental or lobar atelectasis was recorded as atelectasis in this study, whereas linear atelectasis was excluded, as it is generally considered a minor radiological finding. Data on these early complications were limited to the 14-day perioperative period and were not included in the 3- or 6-month functional follow-up assessments.

The following perioperative outcomes were recorded: operative time, intraoperative blood loss, duration of postoperative air leak, cumulative postoperative drainage volume, chest tube duration, incidence of chest tube drainage >200 mL, and length of postoperative hospital stay.

#### 2.3.4 The change in bronchial angles measurement

**Figure 1:**
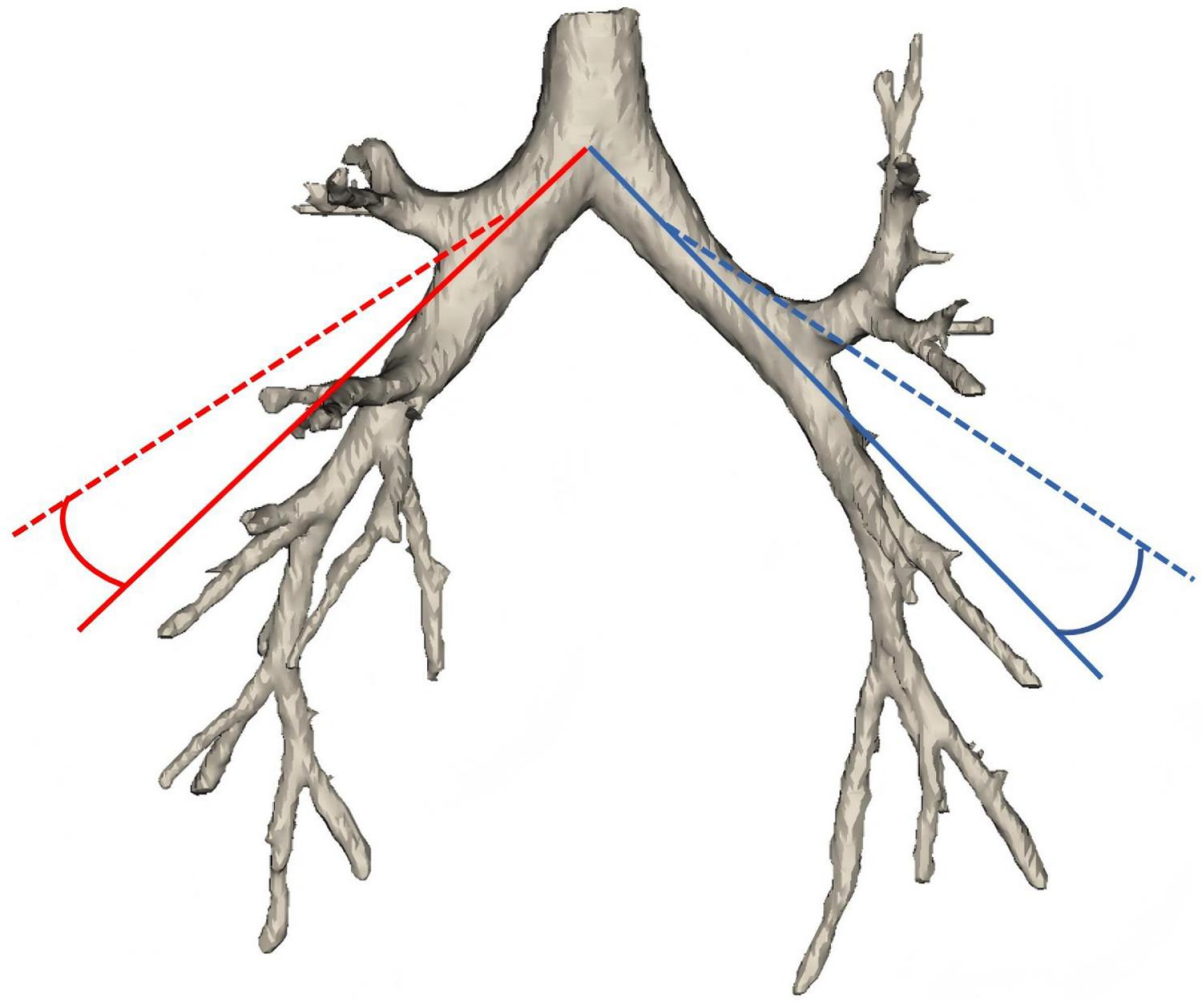
The change in bronchial angle is defined as the preoperative angle minus the postoperative angle. The solid red line represents the axis of the right main bronchus before surgery, while the dashed red line indicates its postoperative axis. The angle between the solid and dashed blue lines corresponds to the difference between the preoperative and postoperative angles. The same principle applies to the left bronchus as indicated by the blue lines.

Bronchial angles were calculated according to methods described in previous studies (10). Three-dimensional reconstructed chest CT images obtained preoperatively and at 3 and 6 months postoperatively were generated using Mimics Research 21.0 software (Materialise, Leuven, Belgium), which was also used to measure changes in bronchial angles. Changes in bronchial angle were measured according to the following principles. (1) For patients undergoing left upper lobectomy, the longitudinal axis of the left main bronchus served as the baseline in the coronal plane, and the angle was defined relative to the axis of the lower lobe bronchus; (2) for those undergoing right upper lobectomy, the baseline was aligned with the axis of the middle lobe bronchus. Angular change was defined as the preoperative angle minus the postoperative angle. All axes were plotted by a radiology resident and subsequently reviewed and adjusted by a senior radiologist (Figure 1).

#### 2.3.5 Changes in lung volume and residual intrathoracic dead space

**Figure 2:**
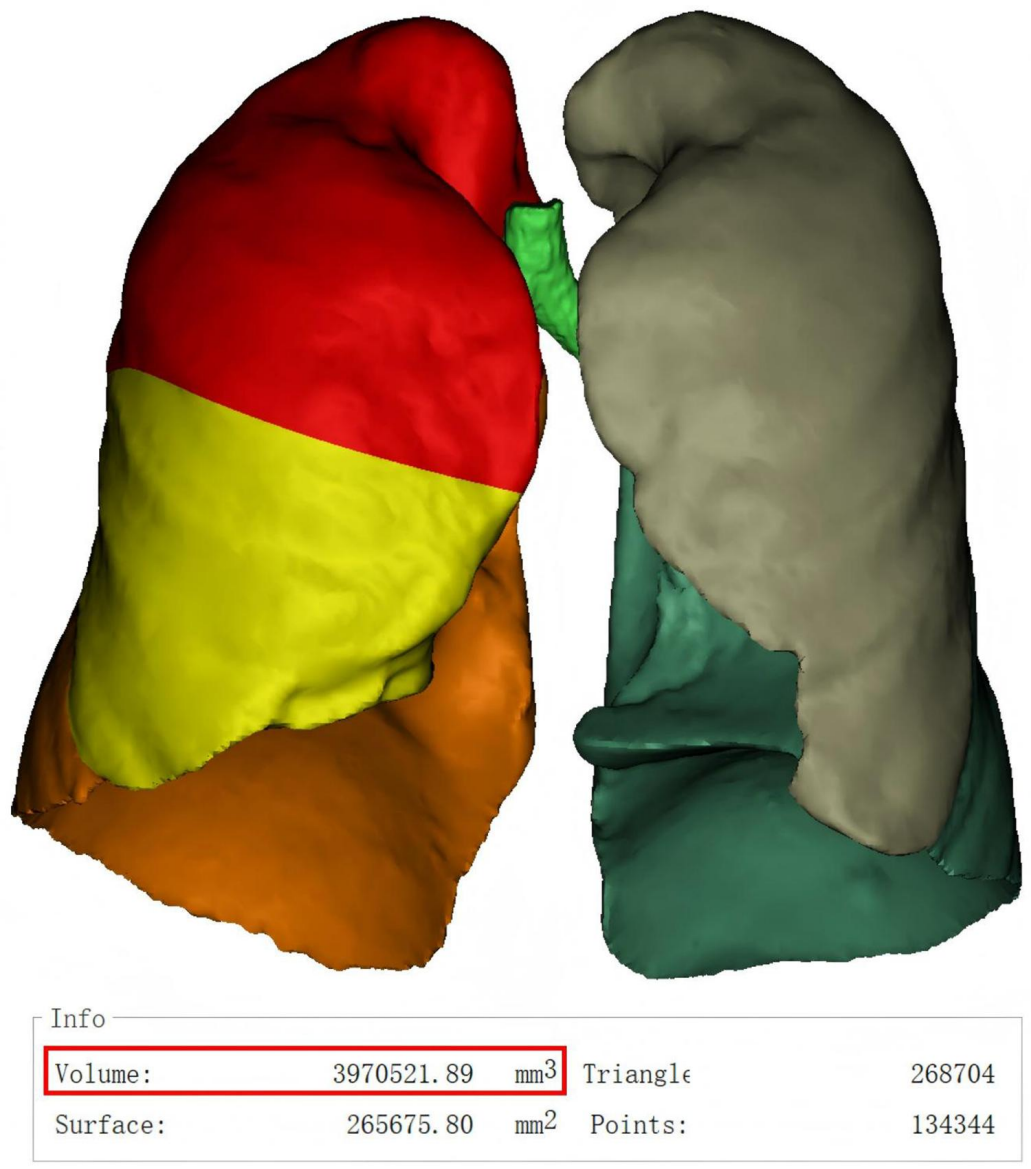
Three-dimensional lung volumetry was conducted semi-automatically with mimics research 21.0 software, utilizing hounsfield unit (HU) values within the range of −1024 to −200.measurements are reported in cubic centimeters (cm^3^), rounded to the first decimal place.as shown in the figure above, the pre-operative total lung volume of this patient was 3970 cm^3^.

Lung volume and residual intrathoracic dead space measurement is a non-invasive, straightforward, and rapid approach for quantifying individual lung volumes and residual intrathoracic dead space using three-dimensional reconstruction of chest CT images. Three-dimensional lung volumetry and residual intrathoracic dead space volumetry were performed semi-automatically using Mimics Research 21.0 software. Lung volumes and residual intrathoracic dead space volumes were recorded in cubic centimeters (cm^3^). Measurements were obtained preoperatively and at 3 and 6 months postoperatively and were documented by the research team (Figure 2).

#### 2.3.6 Evaluation of persistent cough in the preoperative and postoperative period

Patients were assessed using the Mandarin Chinese version of the Leicester Cough Questionnaire (LCQ-MC), which evaluates physical, psychological, and social domains. The questionnaire consists of 19 items, each scored on a 7-point Likert scale, with higher scores indicating less severe cough (16). Domain scores were calculated as the mean of items within each domain (range, 1–7), and the total score was obtained by summing the three domain scores (range, 3–21) (16). All patients completed the LCQ-MC preoperatively and postoperatively under the supervision of two trained medical staff members. Postoperative cough severity was assessed using a visual analogue scale (VAS), with a score ≥60 mm on a 0–100 mm scale defined as clinically significant cough (17).

Data on lung function tests at 3 and 6 months and LCQ-MC scores were extracted from a prospectively maintained institutional database, as these assessments are routinely collected as part of the standard postoperative follow-up protocol in our department.

#### 2.3.7 Statistical analyses

Statistical analyses were performed using SPSS version 27.0 (SPSS Inc., Chicago, IL, USA). Continuous variables are presented as mean ± standard deviation, whereas categorical variables are presented as counts and percentages. The chi-square test, Fisher’s exact test, or Student’s *t*-test was used to compare differences between groups, as appropriate. A two-sided *P* value <0.05 was considered statistically significant. Given the exploratory nature of this study and the evaluation of several prespecified, clinically correlated outcomes (e.g., bronchial angle, lung volume, and pulmonary function), *P* values are reported without formal adjustment for multiple comparisons. These results should be interpreted with caution, particularly those with *P* values close to the significance threshold. When interpreting these results, especially for those with *P*-values close to the significant threshold, this background should be taken into account.

## 3 Result

### 3.1 Patient demographics and perioperative clinical characteristics

**Figure 3:**
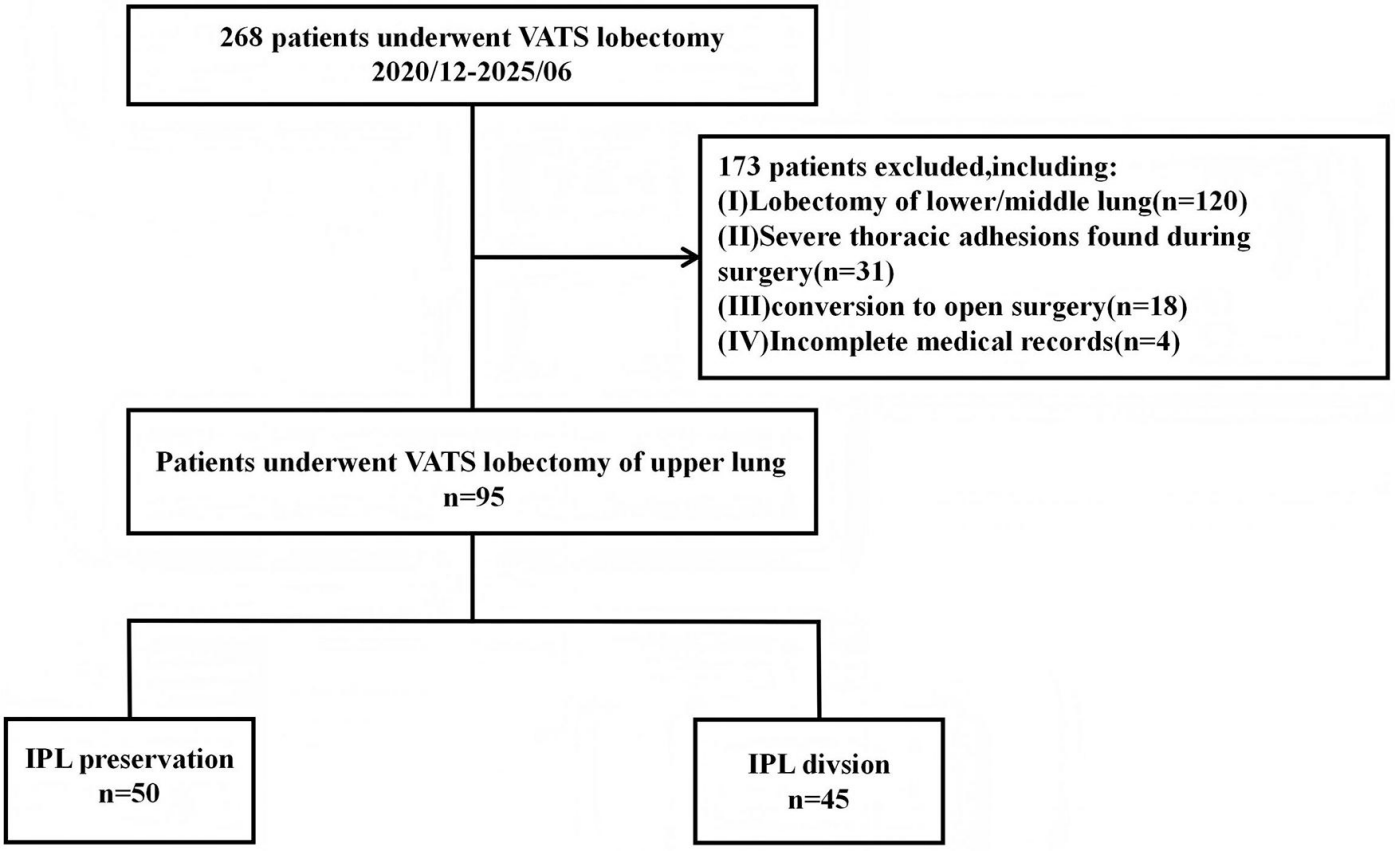
Flowchart of patient selection.

In this retrospective study, a total of 95 patients were enrolled according to the eligibility criteria and were divided into two groups: the IPL-preservation group (Group P, *n* = 50) and the IPL-division group (Group D, *n* = 45). The mean ages were 57.19 ± 11.00 years in Group P and 55.63 ± 12.70 years in Group D, with no statistically significant difference between groups. No statistically significant differences were observed between groups in sex, body mass index, height, weight, smoking status, surgical site, pathological stage, pathological type, preoperative forced expiratory volume in 1 s (FEV1%), preoperative diffusing capacity of the lung for carbon monoxide (DLCO), or preoperative comorbidities (Table 1). The patient selection process is illustrated in Figure 3.

**Table 1:** Baseline characters of patients between two group (pre-op).

Variables	Group P (*n* = 50)	Group D (*n* = 45)	*P*-value
Age (years)	57.19 ± 11.00	55.63 ± 12.70	0.525
Sex			0.926
Male	16	14	
Female	34	31	
BMI (kg/m^2^)	23.65 ± 3.17	23.59 ± 2.32	0.905
Smoking			0.970
Current	13/50 (26.0)	11/45 (24.4)	
Previous	6/50 (12.0)	5/45 (11.1)	
Never	31/50 (62.0)	29/45 (64.4)	
OP side			
Right	31/50 (62.0)	27/45 (60.0)	
Left	19/50 (38.0)	18/45 (40.0)	
Pathological cancer stage			0.576
I	24/50 (48.0)	24/45 (53.3)	
II	18/50 (36.0)	17/45 (37.8)	
III	8/50 (16.0)	4/45 (8.9)	
IV	0 (0.0)	0 (0.0)	
Histology			0.452
Adenocarcinoma	46/50 (92.0)	42/45 (93.3)	
Squamous	4/50 (8.0)	2/45 (4.4)	
Other	0 (0.0)	1/45 (2.2)	
Pulmonary function			
FEV1 (%)	96.38 ± 15.20	98.64 ± 15.94	0.481
DLCO (%)	99.51 ± 14.55	100.54 ± 12.80	0.717
Comorbidity			
Hypertension	12/50 (24.0)	11/45 (24.4)	0.960
Diabetes	5/50 (10.0)	6/45 (13.3)	0.612
Coronary artery disease	6/50(12.0)	4/45(8.9)	0.622

Data are expressed as n (%) or mean ± standard deviation.

FEV1%, forced expiratory volume in 1 s; DLCO, diffusion capacity of the lung for carbon monoxide.

### 3.2 Complications and other clinical data

A comparative analysis of postoperative complications and perioperative outcomes between the two groups is presented in Table 2. Analyses were performed for the overall cohort and further stratified by the side of resection (right or left). No statistically significant differences were observed between groups in the incidence of postoperative complications. Specifically, rates of atelectasis (6.0% vs. 11.1%, *P* = 0.370), pulmonary infection (22.0% vs. 28.9%, *P* = 0.440), atrial fibrillation (6.0% vs. 4.4%, *P* = 0.735), pleural effusion (24.0% vs. 20.0%, *P* = 0.639), and subcutaneous emphysema (22.0% vs. 24.4%, *P* = 0.778) were comparable between groups. Key perioperative and recovery outcomes also showed no significant between-group differences. Although operative time was slightly shorter in the IPL-preservation group (87.95 ± 27.86 vs. 93.45 ± 28.41 min), the difference was not statistically significant (*P* = 0.343). All other outcomes—including intraoperative blood loss, duration of postoperative air leak, cumulative postoperative drainage volume, chest tube duration, days with chest tube drainage >200 mL, and length of postoperative hospital stay—also showed no significant differences (all *P* > 0.05; Table 2).

**Table 2:** Comparison of complications and other clinical data.

Variables	Group P (*n* = 50)	Group D (*n* = 45)	*P*-value
Overall			
Pulmonary atelectasis	3 (6.0%)	5 (11.1%)	0.370
Pulmonary infection	11 (22.0%)	13 (28.9%)	0.440
Atrial fbrillation	3 (6.0%)	2 (4.4%)	0.735
Pleural effusion	12 (24.0%)	9 (20.0%)	0.639
Subcutaneous emphysema	11 (22.0%)	11 (24.4%)	0.778
Operation time (min)	87.95 ± 27.86	93.45 ± 28.41	0.343
Intraoperative bleeding (mL)	61.76 ± 21.82	64.06 ± 23.02	0.618
Postoperative air leak duration (d)	2.22 ± 1.00	2.44 ± 1.34	0.354
Cumulative postoperative drainage (mL)	520.99 ± 72.25	521.20 ± 80.21	0.989
Chest tube duration (d)	6.59 ± 1.71	6.96 ± 1.58	0.273
Chest tube drainage >200 mL (d)	3.48 ± 1.20	3.73 ± 1.52	0.380
Postoperative hospital stay (d)	7.43 ± 1.72	7.35 ± 1.79	0.808
Right	*n* = 31	*n* = 29	
Pulmonary atelectasis	2 (6.4%)	3 (10.3%)	0.586
Pulmonary infection	6 (19.3%)	7 (24.1%)	0.653
Atrial fbrillation	1 (3.2%)	1 (3.4%)	0.962
Pleural effusion	8 (25.8%)	5 (17.2%)	0.421
Subcutaneous emphysema	4 (12.9%)	6 (20.7%)	0.419
Operation time (min)	86.93 ± 29.47	92.67 ± 27.15	0.473
Intraoperative bleeding (mL)	58.18 ± 21.63	61.37 ± 20.61	0.561
Postoperative air leak duration (d)	2.16 ± 1.07	2.38 ± 1.23	0.576
Cumulative postoperative drainage (mL)	532.38 ± 68.45	512.23 ± 77.34	0.290
Chest tube duration (d)	6.65 ± 1.68	6.82 ± 1.47	0.680
Chest tube drainage >200 mL (d)	3.46 ± 1.22	3.71 ± 1.48	0.473
Postoperative hospital stay (d)	7.57 ± 1.90	7.43 ± 1.71	0.768
Left	*n* = 19	*n* = 16	
Pulmonary atelectasis	1 (5.3%)	2 (12.5%)	0.446
Pulmonary infection	5 (26.3%)	6 (37.5%)	0.478
Atrial fbrillation	2 (10.5%)	1 (6.3%)	0.653
Pleural effusion	4 (21.1%)	4 (25.0%)	0.782
Subcutaneous emphysema	7 (36.8%)	5 (31.3%)	0.728
Operation time (min)	89.61 ± 25.70	94.88 ± 31.42	0.589
Intraoperative bleeding (mL)	67.61 ± 21.39	68.94 ± 26.87	0.872
Postoperative air leak duration (d)	2.26 ± 0.97	2.48 ± 1.43	0.476
Cumulative postoperative drainage (mL)	502.39 ± 76.24	537.45 ± 84.58	0.206
Chest tube duration (d)	6.49 ± 1.80	7.22 ± 1.79	0.237
Chest tube drainage >200 mL (d)	3.53 ± 1.20	3.77 ± 1.63	0.619
Postoperative hospital stay (d)	7.20 ± 1.40	7.20 ± 1.98	0.999

Data are expressed as *n* (%) or mean ± standard deviation.

For right-sided resections, complication rates were similar between Group P (*n* = 31) and Group D (*n* = 29), including atelectasis (6.4% vs. 10.3%, *P* = 0.586) and pulmonary infection (19.3% vs. 24.1%, *P* = 0.653). Operative time (86.93 ± 29.47 vs. 92.67 ± 27.15 min, *P* = 0.473) and all other continuous outcomes did not differ significantly between groups. Findings for left-sided resections were consistent, with no significant between-group differences in complications (pulmonary infection: 26.3% vs. 37.5%, *P* = 0.478) or perioperative outcomes (operative time: 89.61 ± 25.70 vs. 94.88 ± 31.42 min, *P* = 0.589) between Group P (*n* = 19) and Group D (*n* = 16) (Table 2).

### 3.3 Change in bronchial angles between two groups

Regarding changes in bronchial angles (Table 3), at 6 months postoperatively, the bronchial angle in the right-sided subgroup was significantly smaller in Group P (45.93 ± 9.14°) than in Group D (52.20 ± 7.27°), with a mean difference of −6.27° (95% CI: −10.49 to −2.05; *P* = 0.005). In the left-sided subgroup, at 3 months postoperatively, the bronchial angle was significantly smaller in Group P (65.78 ± 6.82°) than in Group D (74.70 ± 10.35°), with a mean difference of −8.92° (95% CI: −14.92 to −2.92; *P* = 0.004). Similarly, at 6 months postoperatively, the bronchial angle in the left-sided subgroup was significantly smaller in Group P (71.78 ± 9.51°) than in Group D (82.61 ± 7.25°), with a mean difference of −10.83° (95% CI: −16.48 to −5.18; *P* < 0.001). In contrast, no significant between-group differences in bronchial angle change were observed after right-sided surgery at 3 months (46.08 ± 6.14° vs. 48.05 ± 7.14°, *P* = 0.256).

**Table 3:** Change in bronchial angles between two groups (°, *x* ± *s*).

Variables	Group P	Group D	MD (95% CI)	*P*-value
Right	*n* = 31	*n* = 29		
Preoperative angle	−25.84 ± 1.46	−26.52 ± 1.47	0.68 (−0.06 to 1.42)	0.079
3-mo postoperative	46.08 ± 6.14	48.05 ± 7.14	−1.97 (−5.34 to 1.40)	0.256
6-mo postoperative	45.93 ± 9.14	52.20 ± 7.27	−6.27 (−10.43 to −2.11)	0.005
Left	*n* = 19	*n* = 16		
Preoperative angle	−10.51 ± 2.49	−11.37 ± 1.74	0.86 (−0.54 to 2.26)	0.251
3-mo postoperative	65.78 ± 6.82	74.70 ± 10.35	−8.92 (−14.85 to −2.99)	0.004
6-mo postoperative	71.78 ± 9.51	82.61 ± 7.25	−10.83 (−16.39 to −5.27)	<0.001

In addition, the greatest observed bronchial angle changes were +28.4° at 3 months and +34.7° at 6 months in Group D, and +19.6° and +24.1°, respectively, in Group P (all in left upper lobectomy cases). A comprehensive review of all 3- and 6-month CT scans revealed no instances of bronchial stenosis, bronchial torsion, or clinically significant kinking in either group.

### 3.4 Change in lung volume and pulmonary function between two groups

For lung volume and pulmonary function (Table 4), no significant differences were observed preoperatively or at 3 months postoperatively (*P* > 0.05). There was no significant difference in intrathoracic residual dead space volume between the two groups at either 3 or 6 months postoperatively (*P* > 0.05). At 6 months after surgery, lung volume was significantly greater in Group P (3615 ± 475 mL) than in Group D (3392 ± 489 mL), with a mean difference of 223.0 mL (95% CI: 27.2–418.8; *P* = 0.027). At the same time point, FEV1% was significantly higher in Group P (73.04 ± 9.36%) than in Group D (69.06 ± 10.11%), with a mean difference of 3.98% (95% CI: 0.01–7.95; *P* = 0.049). DLCO was also significantly higher in Group P (80.82 ± 10.35%) than in Group D (76.06 ± 11.08%), with a mean difference of 4.76% (95% CI: 0.41–9.11; *P* = 0.033).

**Table 4:** Change in lung volume and pulmonary function between two groups.

Variables	Group P (*n* = 50)	Group D (*n* = 45)	MD (95% CI)	*P*-value
Preoperative				
Lung volume (mL)	3962 ± 1384	4067 ± 1094	−105.0 (−610.1 to 400.1)	0.685
FEV1 (%)	96.38 ± 15.20	98.63 ± 15.94	−2.25 (−8.59 to 4.09)	0.481
DLCO (%)	99.51 ± 14.55	100.53 ± 12.80	−1.02 (−6.58 to 4.54)	0.717
3-mo postoperative				
Lung volume (mL)	3359 ± 865	3164 ± 737	195.0 (−131.7 to 521.7)	0.241
Residual dead space (mL)	132.5 ± 42.8	128.4 ± 45.1	4.1(−13.7 to 21.9)	0.652
FEV1 (%)	69.74 ± 8.83	69.57 ± 10.26	0.17 (−3.72 to 4.06)	0.929
DLCO (%)	76.09 ± 8.04	74.80 ± 13.12	1.29 (−3.11 to 5.69)	0.569
6-mo postoperative				
Lung volume (mL)	3615 ± 475	3392 ± 489	223.0 (27.2 to 418.8)	0.027
Residual dead space (mL)	95.6 ± 31.4	92.8 ± 33.7	2.8(−10.5 to 16.1)	0.679
FEV1 (%)	73.04 ± 9.36	69.06 ± 10.11	3.98 (0.01 to 7.95)	0.049
DLCO (%)	80.82 ± 10.35	76.06 ± 11.08	4.76 (0.41 to 9.11)	0.033

FEV1%, forced expiratory volume in 1 s; DLCO, difusing capacity of the lung for carbon monoxide.

### 3.5 Comparison of LCQ-MC scores between the Two groups before and after surgery

Regarding LCQ-MC scores (Table 5), no significant between-group differences were observed preoperatively. Postoperatively, the LCQ-MC physical score was significantly higher in Group P (5.91 ± 0.50) than in Group D (5.42 ± 0.51), with a mean difference of 0.49 (95% CI: 0.28–0.70; *P* < 0.001). The psychological score was significantly higher in Group P (5.87 ± 0.53) than in Group D (5.56 ± 0.52), with a mean difference of 0.31 (95% CI: 0.10–0.52; *P* < 0.001). The social score was also significantly higher in Group P (5.89 ± 0.56) than in Group D (5.52 ± 0.55), with a mean difference of 0.37 (95% CI: 0.14–0.60; *P* = 0.021). Finally, the postoperative total LCQ-MC score was significantly higher in Group P (17.70 ± 1.72) than in Group D (16.98 ± 1.69), with a mean difference of 0.72 (95% CI: 0.03–1.41; *P* = 0.042).

**Table 5:** The mean LCQ-MC score of two group.

Variables	LCQ-MC score	Group P (*n* = 50)	Group D (*n* = 45)	MD (95% CI)	*P*-value
Preoperative	Physical	6.89 ± 0.23	6.86 ± 0.26	0.03 (−0.07 to 0.13)	0.903
	Psychological	6.76 ± 0.31	6.71 ± 0.33	0.05 (−0.08 to 0.18)	0.943
	Social	6.75 ± 0.26	6.81 ± 0.28	−0.06 (−0.17 to 0.05)	0.510
	Total	20.50 ± 0.70	20.39 ± 0.95	0.11 (−0.23 to 0.45)	0.664
Postoperative	Physical	5.91 ± 0.50	5.42 ± 0.51	0.49 (0.28 to 0.70)	<0.001
	Psychological	5.87 ± 0.53	5.56 ± 0.52	0.49 (0.28 to 0.70)	<0.001
	Social	5.89 ± 0.56	5.52 ± 0.55	0.37 (0.14 to 0.60)	0.021
	Total	17.70 ± 1.72	16.98 ± 1.69	0.72 (0.03 to 1.41)	0.042

LCQ-MC, Mandarin Chinese version of the Leicester Cough Questionnaire.

### 3.6 Subgroup analysis of key outcomes in group P

Station 9 lymph nodes were removed in 7 of 50 patients (14%) in Group P without IPL dissection. No statistically significant differences were observed in any primary endpoints, including changes in bronchial angle at 3 months (*P* = 0.812) and 6 months (*P* = 0.745), lung volume at 6 months (*P* = 0.683), FEV1% at 6 months (*P* = 0.521), DLCO at 6 months (*P* = 0.597), or total LCQ-MC score (*P* = 0.439) (Table 6).

**Table 6:** Subgroup analysis of key outcomes in group P according to station 9 lymph node dissection status.

Variables	Station 9 dissected (*n* = 7)	Station 9 not dissected (*n* = 43)	*P*-value
Preoperative angle (°)	−10.9 ± 2.7	−10.4 ± 2.4	0.712
3-mo postoperative angle (°)	66.4 ± 7.5	65.9 ± 6.7	0.819
6-mo postoperative angle (°)	72.8 ± 10.2	71.6 ± 9.3	0.746
Preoperative			
Lung volume (mL)	3940 ± 1420	3975 ± 1370	0.891
FEV1 (%)	95.8 ± 16.1	96.5 ± 15.0	0.874
DLCO (%)	98.7 ± 15.2	99.7 ± 14.4	0.802
3-mo postoperative			
Lung volume (mL)	3330 ± 910	3365 ± 850	0.835
FEV1 (%)	69.4 ± 9.8	69.9 ± 8.7	0.821
DLCO (%)	75.2 ± 10.8	76.3 ± 7.6	0.678
6-mo postoperative			
Lung volume (mL)	3580 ± 520	3620 ± 470	0.683
FEV1 (%)	71.9 ± 10.4	73.3 ± 9.2	0.521
DLCO (%)	79.6 ± 11.8	81.1 ± 10.1	0.597
LCQ-MC score (Total)			
Preoperative	20.38 ± 0.82	20.47 ± 0.69	0.724
Postoperative	17.42 ± 1.88	17.75 ± 1.68	0.439

## 4 Discussion

The clinical value of IPL division during thoracoscopic upper lobectomy remains controversial, owing to conflicting evidence regarding its benefits and potential complications. Several studies have suggested that IPL release during upper lobectomy may facilitate lower-lobe mobilization and re-expansion, potentially reducing postoperative complications such as atelectasis and pleural effusion (10, 12, 18–20). However, others have raised concerns that IPL division may have the opposite effect, potentially increasing the risk of bronchial torsion and bronchial stricture. Such changes may manifest as chronic cough and dyspnea and may ultimately contribute to impaired pulmonary function (21, 22). A questionnaire-based study in Japan reported that 69% of hospital-based surgeons tend to preserve the IPL during upper lobectomy. These surgeons believe that preserving IPL integrity may reduce postoperative bronchial torsion and stricture. However, they also acknowledge that this approach may increase the risk of postoperative pleural effusion and pleural infection (23).

Our findings are consistent with a recent multicenter propensity score–matched analysis (9). Both studies confirm that IPL division leads to more pronounced bronchial remodeling. However, important methodological differences exist: Campisi et al. employed a larger, multicenter, propensity-matched design focused primarily on short-term clinical endpoints, whereas our single-center study, although smaller and unmatched, incorporated 3D-CT-based lung volumetry, serial pulmonary function tests, and validated LCQ-MC cough scores at fixed 3- month and 6-month intervals. These more sensitive functional and patient-reported endpoints revealed modest but statistically significant advantages of IPL preservation in lung volume recovery, FEV1% preservation, and cough-related quality of life—benefits that may only become apparent beyond the immediate postoperative period captured in Campisi et al. Collectively, the two studies suggest that while IPL division does not increase short-term morbidity, preservation may confer subtle long-term anatomical and functional benefits that warrant further investigation in prospective randomized trials.

Traditionally, IPL preservation during upper lobectomy has been thought to increase postoperative complications, including apical dead space, atelectasis, and pleural effusion (23). Advocates of IPL division argue that IPL preservation may increase postoperative dead space, which could contribute to pleural effusion and other complications. However, three-dimensional (3D) CT volumetric reconstruction in this study revealed no significant differences in residual dead space volume between the preservation and division groups at 3 or 6 months postoperatively. Compared with traditional imaging assessments reported by Matsuoka et al. (5), our volumetric measurement approach provides greater precision and reliability. This method enables high-resolution, anatomically precise quantification of extrapulmonary air space. These findings indicate that IPL preservation does not impede pleural cavity obliteration in Asian populations. Nevertheless, as Western populations typically exhibit larger average thoracic volumes, further investigation into ethnic variations is warranted to clarify these clinical implications. In the present study, the incidence of postoperative complications—including atelectasis, pulmonary infection, atrial fibrillation, and subcutaneous emphysema—did not differ significantly between Group P and Group D, consistent with previous reports. Although Group D required an additional procedural step (IPL division) and showed longer operative time and greater intraoperative blood loss, these differences did not reach statistical significance. This may reflect the surgeons’ extensive experience with VATS. To date, no studies have reported that IPL division prevents pleural fluid accumulation. In the present study, pleural fluid accumulation was assessed using chest tube duration, total postoperative drainage volume, and the number of days with drainage >200 mL. No significant between-group differences were observed for these measures. Overall, Group D tended to have longer durations of air leak and chest tube placement, although these differences were not statistically significant. This pattern may relate to our institutional practice of early pleurodesis for postoperative air leak. Therefore, days with drainage >200 mL may be a more informative indicator than chest tube duration, because chest tube removal depends not only on drainage volume but also on the presence of air leak.

Some studies have suggested that IPL preservation may reduce the incidence of bronchial distortion and stenosis but may increase the risk of pleural effusion and infection (23). In the present study, however, no significant between-group differences were observed in pulmonary infection, pleural drainage volume, drainage duration, or prolonged air leak. These findings indicate that IPL preservation did not have a significant impact on these outcomes.

Another major concern regarding intraoperative IPL division is alteration of the bronchial angle. Studies have shown that approximately 90% of patients recover pulmonary function to expected levels within 3–6 months after surgery (24). This recovery is attributed to compensatory adaptation and appropriate bronchial remodeling. However, excessive remodeling may cause bronchial deformation, which could in turn impair postoperative compensation. Multiple studies have reported that IPL division may increase the risk of bronchial angulation relative to the tracheal axis, bronchial torsion, airflow disturbance, and respiratory dysfunction, potentially related to residual lung atelectasis. In this study, the greatest bronchial angle change was 88° in Group D at 6 months. The most extreme case occurred following left upper lobectomy. However, no instances of bronchial stenosis, bronchial torsion, or clinically significant kinking were observed in either group. Nevertheless, because excessive bronchial torsion is a key precipitating factor for bronchial stenosis and obstruction, bronchial angle measurement may still be clinically relevant. In patients undergoing right upper lobectomy, Group D showed a greater change in bronchial angle at 6 months (45.93 ± 9.14° vs. 52.20 ± 7.27°, *P* = 0.005). In contrast, in patients undergoing left upper lobectomy, the between-group difference was evident as early as 3 months (65.78 ± 6.82° vs. 74.70 ± 10.35°, *P* = 0.004) and was more pronounced at 6 months (71.78 ± 9.51° vs. 82.61 ± 7.25°, *P* < 0.001). These findings may relate to anatomical differences between the left and right main bronchi. The left main bronchus is longer and more horizontal than the right, which may make it more susceptible to displacement as the lower lobe shifts postoperatively. By contrast, after right upper lobectomy, the right middle lobe may provide partial structural support, and the remaining lung may be anatomically more stable, potentially delaying the emergence of this difference.

By reducing functional lung parenchyma, lobectomy may cause persistent declines in pulmonary function, with potential consequences for quality of life and prognosis (25, 26). Compared with lower lobectomy, upper lobectomy may be associated with greater postoperative loss of pulmonary function (27). IPL preservation during VATS lobectomy may influence postoperative pulmonary function and lung volume. In the present study, lung volume, FEV1%, and DLCO did not differ significantly between groups at 3 months (28). At 6 months, Group P had higher FEV1% and DLCO than Group D. Lung volume was also significantly greater in Group P than in Group D at 6 months. Potential mechanisms include increased bronchial angulation after IPL division, which may predispose to bronchial narrowing and reduced ventilation and gas exchange. In addition, elevation of the lower lobe and diaphragm may reduce thoracic volume and lung volume. By tethering the residual lung, the IPL may help maintain bronchial alignment and reduce the risk of bronchial rotation and traction-related distortion. We hypothesize that this tethering effect may support bronchial remodeling during postoperative lung re-expansion.

The etiology and mechanisms of persistent cough after lobectomy remain incompletely understood, and alterations in the anatomy and physiology of the residual bronchus on the operated side may represent an important contributing factor. Some studies suggest that during upper lobectomy, IPL division may exacerbate bronchial deformation, torsion, and stenosis, thereby aggravating postoperative persistent cough (18, 29). Notably, although Group P showed a greater improvement in cough than Group D at 1 month postoperatively, cough symptoms persisted, albeit with a relatively reduced impact on quality of life.

Although the differences in lung function and cough scores at 6 months reached statistical significance, their absolute magnitudes warrant careful interpretation in terms of clinical relevance. The minimal clinically important difference (MCID) for the LCQ-MC is typically defined as 1.3 units. In this study, the difference in total LCQ-MC scores between Group P and Group D was 0.72, which falls below this threshold. Similarly, the absolute differences in FEV1% (3.98%) and DLCO (4.76%) were modest. However, the clinical relevance of IPL preservation may lie in the combined effect of these outcomes; the consistent trend toward improved bronchial geometry, greater lung volume, and better cough scores suggests a more favorable physiological recovery environment. Even if individual parameters do not reach the MCID, the cumulative effect of avoiding subclinical impairments across multiple domains may lead to a perceptible improvement in overall postoperative quality of life. Although our findings suggest that IPL preservation may confer advantages for patients undergoing VATS upper lobectomy, clinical decision-making should carefully balance other surgical considerations. For example, IPL division may be necessary in some cases to improve surgical exposure or manage pathological conditions such as pleural adhesions. Moreover, in patients with enlarged lower mediastinal lymph nodes, IPL division may be required to facilitate more comprehensive lymph node dissection. Therefore, surgical decisions should be individualized according to patient-specific anatomy, operative goals, and potential risks. This individualized approach aims to optimize surgical outcomes while preserving the accuracy of postoperative pathological staging. Previous studies have shown that routine dissection of station 9 lymph nodes may not be necessary, particularly in patients with low T stage, upper or middle lobe tumors, or no intrapulmonary lymph node metastasis (30). Therefore, station 9 lymph nodes were dissected without IPL delineation only when involvement was confirmed or strongly suspected. In this study, station 9 lymph nodes were dissected in 14% of Group P cases without IPL division. Subgroup analysis demonstrated no significant impact on bronchial angle, lung volume, pulmonary function, or cough-related quality of life, suggesting that limited, node-focused dissection did not compromise ligament integrity or introduce bias into the main findings. These findings support the observed advantages of IPL preservation. Future studies are warranted to evaluate more extensive station 9 lymph node dissection.

Several limitations should be acknowledged. The decision to dissect or preserve the inferior pulmonary ligament was at the surgeon’s discretion without predefined criteria, introducing potential selection bias. Although baseline characteristics were comparable, the retrospective design and lack of adjustment methods (e.g., propensity score matching or multivariable regression) mean residual confounding cannot be excluded. The relatively small sample size also limited stable multivariable and subgroup analyses, particularly for 6-month outcomes. In addition, no formal correction for multiple testing was applied despite multiple comparisons, increasing the risk of Type I error—especially for results near the 0.05 threshold. Although consistent effect directions across several parameters at 6 months suggest a possible true association, these findings should be considered exploratory and require confirmation in larger prospective studies. In practice, all procedures were performed by the same surgical team using standardized techniques and instruments. Therefore, caution is warranted when extrapolating these findings to other centers. Future studies with larger sample sizes and prospective designs should incorporate more robust statistical methods to further validate the association between IPL management and postoperative functional outcomes.

## 5 Conclusion

In conclusion, current evidence does not demonstrate a clear benefit of IPL division. In contrast, IPL division may be associated with reduced postoperative lung volume, impaired recovery of pulmonary function, excessive displacement of the residual bronchus, and chronic cough. Therefore, the potential adverse effects of IPL division during TUL in patients with lung cancer warrant careful consideration.

## Data Availability

The original contributions presented in the study are included in the article/Supplementary Material, further inquiries can be directed to the corresponding author.
